# Plasmepsin-like Aspartyl Proteases in *Babesia*

**DOI:** 10.3390/pathogens10101241

**Published:** 2021-09-26

**Authors:** Pavla Šnebergerová, Pavla Bartošová-Sojková, Marie Jalovecká, Daniel Sojka

**Affiliations:** 1 Institute of Parasitology, Biology Centre, Academy of Sciences of the Czech Republic, Branišovská 1160/31, CZ-37005 České Budějovice, Czech Republic; pavla.snebergerova@paru.cas.cz (P.Š.); bartosova@paru.cas.cz (P.B.-S.); 2 Faculty of Science, University of South Bohemia in České Budějovice, Branišovská 1760c, CZ-37005 České Budějovice, Czech Republic; jalovecka@prf.jcu.cz

**Keywords:** aspartyl protease, plasmepsin, apicomplexa, piroplasmida, *Babesia*

## Abstract

Apicomplexan genomes encode multiple pepsin-family aspartyl proteases (APs) that phylogenetically cluster to six independent clades (A to F). Such diversification has been powered by the function-driven evolution of the ancestral apicomplexan AP gene and is associated with the adaptation of various apicomplexan species to different strategies of host infection and transmission through various invertebrate vectors. To estimate the potential roles of *Babesia* APs, we performed qRT-PCR-based expressional profiling of *Babesia microti* APs (BmASP2, 3, 5, 6), which revealed the dynamically changing mRNA levels and indicated the specific roles of individual BmASP isoenzymes throughout the life cycle of this parasite. To expand on the current knowledge on piroplasmid APs, we searched the EuPathDB and NCBI GenBank databases to identify and phylogenetically analyse the complete sets of APs encoded by the genomes of selected *Babesia* and *Theileria* species. Our results clearly determine the potential roles of identified APs by their phylogenetic relation to their homologues of known function—*Plasmodium falciparum* plasmepsins (PfPM I–X) and *Toxoplasma gondii* aspartyl proteases (TgASP1–7). Due to the analogies with plasmodial plasmepsins, piroplasmid APs represent valuable enzymatic targets that are druggable by small molecule inhibitors—candidate molecules for the yet-missing specific therapy for babesiosis.

## 1. Introduction

Babesiosis (also known as piroplasmosis) is a malaria-like disease caused by the parasites from the genus *Babesia* of the apicomplexan order Piroplasmida. This order was initially represented by three separated lineages—*Babesia*, *Theileria*, and *Cytauxzoon*—but more recent phylogenetic analyses have indicated approximately six lineages of Piroplasmida, out of which the approximately 100 *Babesia* species are represented in at least three distinct clades [1]. Analogously to their malaria-causing relatives (genus *Plasmodium*, order Haemosporidia), *Babesia* parasites are transmitted to their vertebrate hosts via a blood-feeding arthropod (tick), where they follow an asexual erythrocytic growth cycle. With the global distribution of ticks and their dynamically changing distribution in recent decades, babesiosis represents an important worldwide veterinary threat, and an emerging risk to humans [2].

Despite routine epidemiological surveillance, babesiosis has long been recognised as an economically important disease affecting livestock, with growing incidence in both domesticated and wildlife animals [3]. Bovine babesiosis, commonly called red water fever, is the economically most important arthropod-transmitted disease affecting cattle, causing mortalities, miscarriages, and decreased meat production [4]. Recently, increased attention has been devoted to the alarming increase in severe "dog babesiosis", caused mainly by *Babesia canis* transmitted by the ornate dog tick *Dermacentor reticulatus*, which has mosaic distribution throughout Europe [5]. Humans are accidental hosts of *Babesia*, but numerous factors indicate human babesiosis as an emerging zoonosis. The clinical features of human babesiosis are similar to malaria, and can be fatal, particularly in the elderly and in immunocompromised individuals [2]. The majority of human infections are reported from the USA, where the principal agent *Babesia microti* is the most commonly transmitted pathogen during blood transfusions [6,7]. Babesiosis is also an emerging problem in Europe, where *Ixodes ricinus*-transmitted *Babesia divergens* is considered the major causative agent of human babesiosis [8]. Incidence rates of human babesiosis are probably underestimated due to misdiagnosis of malaria in the overlapping distribution areas of these parasitic diseases [9].

Protection against bovine babesiosis is mostly based on the debatable vaccination of young cattle with attenuated parasites [10]. Treatment of human babesiosis is non-specific and relies on the combination of antimalarial drugs and antibiotics, such as atovaquone and azithromycin [6]. However, a remarkable increase in parasite resistance, together with significant numbers of relapsed immunocompromised and asplenic individuals [11], has made this widely used treatment regime less effective [12]. Alternative therapy with clindamycin and quinine is toxic and has never been tested in clinical trials. Reports of babesiosis within new geographical regions, as well as identifications of new *Babesia* species as agents of severe human disease, suggest rapid changes in *Babesia* spp. epidemiology, and make human babesiosis a serious public health concern [2]. Hence, novel approved drugs and veterinary control strategies based on *Babesia*-specific molecular targets are highly desirable [10,12].

Parasite-derived proteolytic enzymes (proteases) have been adapted for various functions connected with the parasitic lifestyle [13]. Aspartyl (aspartic, aspartate) proteases use an activated water molecule bound to one or more aspartate residues for catalysis of their peptide substrate. The peptidase database MEROPS [14] identifies five clans of aspartic proteases (AA, AC, AD, AE, and AF), each representing an independent evolution of the same active site and mechanisms. Proteases in the clan AA are either bilobed (family A1, the pepsin family) or homodimeric (all other families in the clan, including the A2 family of retropepsins) [15]. Each lobe consists of a single domain with a closed beta-barrel, and each lobe contributes one aspartate to form the active site. The monomers (retroviruses, retrotransposons, and badnaviruses) are structurally related to one lobe of the pepsin molecule. Due to their ability of specific cleavage within protein bonds, pepsin family (A1) aspartyl proteases (APs) have been evolutionary selected and adapted for unique cellular and physiological roles [16]. Apicomplexa, in contrast to metazoan parasites, have used rapid evolution of the single ancestral A1 protease, resulting in multiple AP-encoding genes being found in their genomes, as best represented by the 7 different APs of *Toxoplasma gondii* (TgASP1–7) [17] and 10 plasmepsin isoenzymes encoded by the genome of *P. falciparum* (PfPM I–X) [18]. The multiple Apicomplexan APs are phylogenetically clustered into six clearly distinguishable clades, tagged A–F. These clades reflect the function-driven evolution and various biological roles of these enzymes within the life cycle of these parasitic organisms [16,17]. Due to their essential roles and affordable selective targetability by small molecule inhibitors, APs have been considered and validated as valuable therapeutic targets [16,19].

In this work, we use the available EuPathDB and NCBI GenBank database records to identify and phylogenetically analyse the yet unrevealed plasmepsin and other apicomplexan AP homologues from selected *Babesia* and *Theileria* species. We estimate the roles of newly identified entries by their phylogenetic clustering to the six clades of apicomplexan APs, as well as their evolutionary relation to *P. falciparum* plasmepsins and *T. gondii* ASP homologues of known functions. Our estimation is supported by the dynamic expression profiling throughout selected life stages of *Babesia microti* using our previously developed *B. microti–Ixodes ricinus*–mouse acquisition model [20].

## 2. Results and Discussion

### 2.1. Expression Profiling of BmASPs Indicate Their Various Roles throughout the Life Cycle of Babesia microti

**Figure 1:**
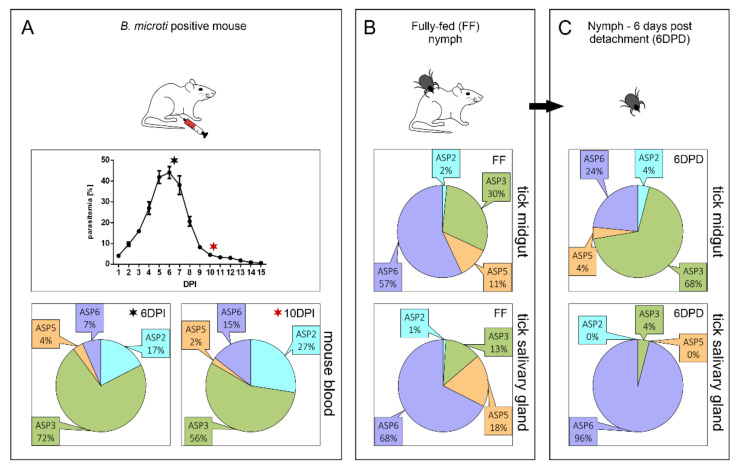
Dynamic expression profile of aspartyl proteases (BmAPs) during the life cycle of *B. microti*. The qPCR results were obtained with cDNA templates prepared from the total RNA isolated: *A*—from mouse blood on the 6th (red asterisk) and 10th (black asterisk) days post parasite injection; *B*—from infected midgut and salivary glands of *I. ricinus* nymphs at fully fed tick stage; *C*—6 days post detachment of *I. ricinus* nymphs from the host. DPI: days post-injection; FF: fully fed stage; 6DPD: 6 days post detachment; ASP2: BmASP2 (BMR1_01G02485, clade B); ASP3: BmASP3a (BmR1_04g07350, clade C); ASP5: BmASP5 (BmR1_04g05270, clade D); ASP6: BmASP6 (BMR1_03g03850, clade E). Relative expression of individual BmASP-encoding mRNAs was counted as percentage ratios of the BmASP mRNA with the highest Ct value (100%) for each cDNA template. The obtained ratios were used to create the pie charts (100% total).

Based on the previously published phylogenetic analysis of parasite cathepsin D-like proteases [16], we initially assessed the expression dynamics of four identified *B. microti* APs (BmASPs) to estimate their potential roles within the life cycle of *B. microti*. This was enabled by the use of our established *B. microti* acquisition model, involving BALB/c mice and *I. ricinus* tick nymphs [20]. Our results clearly show *B. microti* ASP3 (BmASP3, BmR1_04g07350) to be the most abundantly expressed AP isoenzyme of the parasite intraerythrocytic stage (Figure 1A). This finding is in line with the phylogenetic clustering of BmASP3 to clade C of apicomplexan APs, together with the *P. falciparum* plasmepsins PfPM IX and PfPM X [21], and *T. gondii* ASP3 [22], which are the apical complex-associated proteases playing essential roles in parasite invasion and egress from the host cell (further discussed in Section 2.2.). Analogously to the clade C plasmepsins, BmASP3 is also expressed in *B. microti* stages developing in the midgut and salivary glands of *I. ricinus* nymphs (Figure 1B,C), indicating the potential involvement of BmASP3 in tick tissue invasion.

*B. microti* ASP6 (BmASP6; BMR1_03g03850) mRNA represents the relatively most abundant AP encoding mRNA (54%) in the gut of fully fed tick nymphs, and its abundance in this tissue remains strong even 6 days post the detachment of tick nymphs from their host (24%) (Figure 1B,C). This indicates the involvement of BmASP6 in *Babesia* zygote development and resembles the mRNA expression profile of the *P. falciparum* orthologue PM VII. This enzyme is present in all sexual stages of *P. falciparum* [23], although the protein first appears in diploid stages upon fertilization, presumably due to regulated protein translation [23]. Since BmASP6 is also expressed in *B. microti* stages developing in the tick midgut 6 days post tick detachment, we hypothesise its potential role during the *B. microti* zygote–kinete stage transition, and the subsequent development of kinetes occurring in the tick midgut wall. Supported by the predominant expression of BmASP6 in the salivary glands of fully fed and detached *I. ricinus* nymphs (Figure 1B,C), BmASP6 presumably plays a role in the dissemination of kinetes to other tick tissues, including salivary glands, and subsequently in the early phase of *B. microti* sporogony. The BmASP6 *T. gondii* orthologue TgASP6 is expressed in sporulated oocyst-containing haploid sporozoites [17]. Since piroplasms do not create oocysts in tick midguts, and sporogony occurs in tick salivary glands, we speculate that BmASP6 might be synthesised as a precursor during early sporogony, and that the enzyme could be catalytically activated upon tick feeding. Mature BmASP6 might thus be involved in the release of sporozoites to tick saliva and/or sporozoite invasion of host red blood cells.

Interestingly, the clade B member *B. microti* ASP2 (BmASP2, BMR1_01G02485) is relatively more expressed in *B. microti* blood stages. This contrasts with its orthologue PfPM VI, which is specifically expressed in the vector stages, and is involved in the correct transition of sporozoites from the oocyst [24]. BmASP2 is relatively lowly expressed in monitored tick stages (Figure 1B,C), while it appears abundant (up to 27%) in host blood (Figure 1A). Thus, BmASP2 might be already produced in gametocytes in host blood, and the protein is first produced upon fertilization in tick midguts due to regulated translation in the same manner as the clade E PfPM VII [23]. Alternatively, it might play a different role during the *B. microti* life cycle than its orthologue PfPM VI [25].

The clade D member *B. microti* ASP5 (BmASP5, BmR1_04g05270), the PEXEL (TEXEL)-cleaving *P. falciparum* PM V [26,27], and *T. gondii* ASP5 [27,28] orthologue, are expressed throughout all three selected timepoints of the *B. microti* life cycle (Figure 1). Since the exported proteins of *B. microti* were proposed to lack the PEXEL-like motifs [29] and their trafficking is most likely mediated via vesicles [30], BmASP5 supposedly plays other roles throughout the parasite life cycle, e.g., in the intracellular trafficking of proteins to specific organelles [31], in the development of gametocytes analogous to PfPM V [32], or in the secretion of proteins interacting with tick tissues during transmission. However, the role of *B. microti* PEXEL processing should not be fully excluded, as only more complex studies on the *B. microti* secretome can fully address this point in the future.

### 2.2. Data-Mining and Phylogenetic Analysis of Piroplasmid APs Reveals the Presence of Multiple AP Isoenzymes Clustering to Several Apicomplexan AP Clades

#### 2.2.1. Clade A

**Figure 2:**
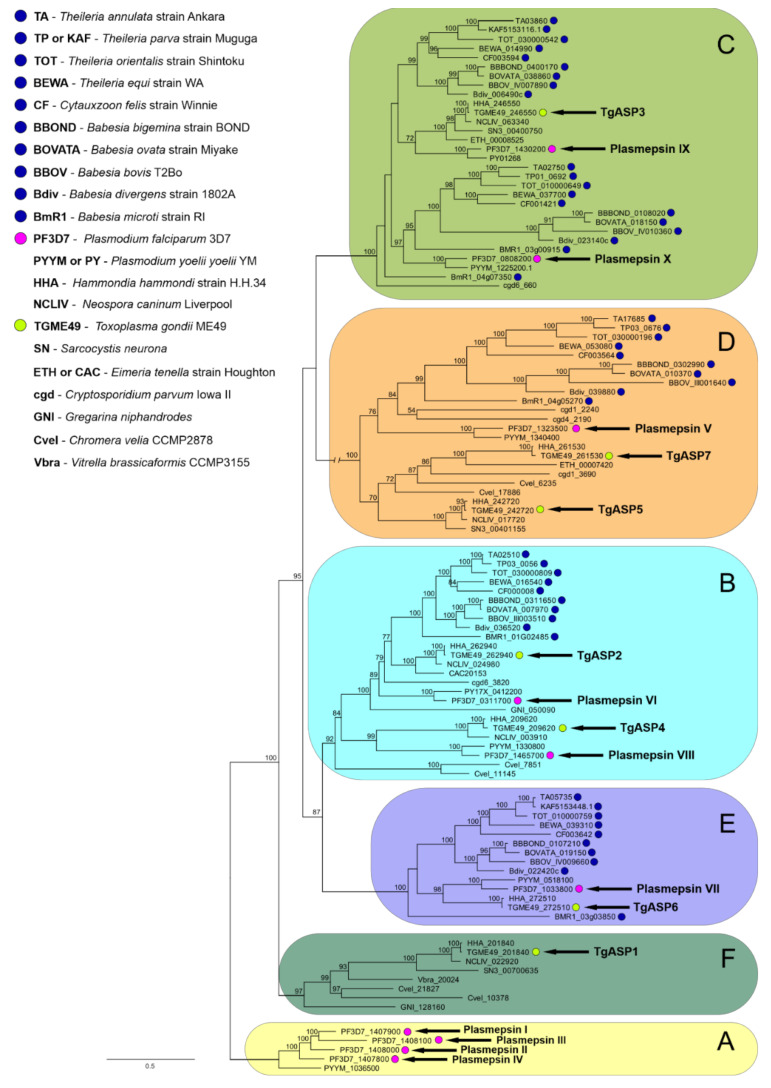
Phylogenetic tree of apicomplexan pepsin family aspartyl proteases (AP). Data-mined APs from selected piroplasmid species cluster into previously determined clades A–F together with their apicomplexan homologues. The image displays the unrooted maximum likelihood phylogenetic tree of 106 selected apicomplexan AP sequences, including those from the basal free-living species *Vitrella brassicaformis* and *Chromera velia;* clades A–F are tagged and highlighted with colours. Sequences were retrieved from EuPathDB and GenBank, and the source organisms are indicated (upper left). Nodal supports were calculated from 1000 bootstrap replicates; those lower than 50 are not depicted. For better orientation within the tree, the 7 *Toxoplasma gondii* ASPs and the 10 *Plasmodium falciparum* plasmepsins are highlighted (arrows: yellow-green and magenta dots, respectively). Data-mined piroplasmid APs are tagged with dark blue dots. Note: The branch leading to clade D was shortened to 25% of its original length for optimal image display. Multiple alignment data are accessible as an online supplementary file at Mendeley Data, link: http://dx.doi.org/10.17632/ds3f2j32ny.1, accessed on 23 September 2021.

Our data search throughout available Piroplasmida sequence databases did not reveal a single AP isoenzyme that would cluster into clade A (Figure 2) represented by the digestive vacuole-residing hemoglobinolytic *Plasmodium falciparum* plasmepsins I, II, and IV (PfPM I, PfPM II, and PfPM IV), or the histo-aspartyl protease (HAP/PfPM III) [33]. This clade has apparently formed under the evolutionary demand to digest host haemoglobin. Although both apicomplexan sister orders Haemosporida and Piroplasmida are obligate intracellular parasites whose propagation strictly depends on nutrients provided by the host cell [34], their feeding mechanisms likely differ, which is also reflected in their classification: *Plasmodium* spp. intraerythrocytic stages digest a substantial amount (up to 80%) of host cell haemoglobin (Hb) [35]. Hb proteolysis releases heme, and globin serves as a source of free amino acids. During Hb digestion within the acidic environment of the food vacuole (FV), clade F plasmepsins tightly cooperate with cysteine proteases—falcipains, metalloprotease falcilysin, and other aminopeptidases that complete the degradation process [36]. The digestive plasmepsins are the most closely related isoenzymes among the 10 *P. falciparum* plasmepsins; they share 50–70% amino acid identity, and their encoding genes are located together on one chromosome [37]. Interestingly, the plasmodial species outside the primate-infecting group of *P. falciparum* encode for a single digestive plasmepsin, analogous to PfPM IV [37]. The heme moiety originating from host Hb does not appear to be metabolised or recycled; instead, it is aggregated and polymerised to the dark pigmented hemozoin (Hz) (the name of the order Haemosporida) [38,39,40].

In contrast to *Plasmodium* spp., piroplasms degrade little if any Hb during host erythrocyte infection, and no pigmented Hz is formed [41,42,43]. Knowledge of the exact routes of nutrient uptake and processing remains rather unclear and is mainly based on electron microscopy observations of trophozoites. The FV formation via cytostome has been observed in *Theileria* spp., where the optical density of this vacuole indicated potential Hb presence [44]. In addition, *T. equi* possibly uses another tubular feeding structure that is formed via the invagination of an erythrocyte plasma membrane enclosing only a minor amount of ingested cytoplasmic material [45]. *Babesia* does not possess a cytostome; thus, the true FV that emerges from its constriction cannot be formed. The ovoid bodies in *Babesia* trophozoites, initially considered to be true FVs, have since been recognized as invaginations of host cell cytoplasm [46]. In *B. microti*—the basal piroplasmid species (*Babesia microti*-like group)—the obscure coiled structure that protrudes from the parasite into the host cytoplasm has been observed and speculated to contain digestive enzymes. Similarly, possible haemoglobin-containing vesicles were also observed from *B. divergens* trophozoites, indicating—yet not confirming—the endocytic uptake of hemoglobin from the cytoplasm of host erythrocytes [47]. The role of hemoglobin invaginations in trophozoites of different species of *Babesia* remains unclear, but could be relevant to the biology of piroplasms, e.g., by limited digestion of hemoglobin providing the essential source of heme to these heme auxotrophic organisms. Moreover, the special organelle of *B. microti* allegedly contains ferritin, which may be used as a nutrient source for *Babesia* [46]. Although piroplasms do not encode direct homologues of Hb-digesting plasmepsins, they do encode several papain-like cysteine proteases [36]. However, these orthologues of hemoglobinolytic *Plasmodium* spp. falcipains have not yet been subjected to biochemical and functional characterization that would reliably validate their contribution to Hb digestion in erythrocyte-residing piroplasms. Thus far, the only evidence of their essential role in the survival of *Babesia* spp. parasites has come from the observation of a hampering effect on *B. bovis* erythrocyte invasion and in vitro replication by cysteine protease inhibitors [48], and from the lowered parasitemia observed in *B. ovis* erythrocyte cultures exposed to antibodies against ovipain-2, the *B. ovis* orthologue of falcipain-2 from *P. falciparum* [49]. The same applies to the falcilysin protein family represented by one or two isoenzymes in *Babesia* and *Theileria*, respectively [36]. A homologue of the *P. falciparum* heme detoxification protein (HDP) has already been identified in *Babesia* and *Theileria*. However, this homologue supposedly plays a different role than in *Plasmodium* spp., since piroplasms do not form the hemozoin pigment [36]. Overall, the molecular basis of feeding and catabolic metabolism in piroplasms, and the involvement of individual aspartyl and cysteine proteases in protein digestion, remain unclear, and should be addressed by future experimental studies.

#### 2.2.2. Clade B

Clade B is an independent cluster originally recognized as the ancestral group of apicomplexan APs by Jean et al. [50]. Some members of this group—for example, the proteases of *T. gondii* (TgASP2 and TgASP4), *Eimeria tenella* (eimepsin), *Cryptosporidium parvum* (EAK89992), and *Theileria annulata* (TA02510)—are predicted to be GPI-anchored, while plasmepsins VI and VIII lack a sufficiently long hydrophobic region at the C-terminus for the GPI anchor prediction [17]. Our analyses confirm the phylogenetic relation of clades B and E (discussed below) and highlight the existence of three subclusters within the clade B. In addition to the basal subgroup represented by the two *Chromera velia* isoenzymes, the two other independent subgroups are represented by plasmepsin VI (PM VI)/TgASP2 and plasmepsin VIII (PM VIII)/TgASP4, respectively (Figure 2). Interestingly, all analysed piroplasmid species encode for a single clade B AP orthologue clustering with the first listed group of PM VI/TgASP2. Analogously to PM VI, no GPI anchor is predicted in this enzyme (data not shown). Clade B member plasmepsins, studied mostly in the rodent malaria parasite *Plasmodium berghei*, are primarily expressed in oocysts and sporozoites—the parasite transmission stages [24,51]. They play a role in midgut sporozoite development. Functional genomic analyses involving gene disruptions did not confirm the essential roles of these enzymes for the blood stages of malaria [24,52], but the *pm vi* gene knockout did affect sporozoite development from oocysts, resulting in an unsuccessful transmission of the parasite through the mosquito vector [24]. The role of the plasmepsin VI piroplasmid orthologues might thus be connected to the penetration and migration of piroplasmid stages through tick tissues, offering a suitable target to develop transmission-blocking therapy. However, our dynamic expression profiling (Figure 1) analysis controversially revealed a higher abundance of BmASP2-encoding mRNA in the blood stages of *B. microti* in comparison to the tick tissue isolates. This might be explained by the expression of clade B piroplasmid APs already encoding mRNA in gametocytes in host blood, and by regulated protein translation of the clade B AP enzyme later in tick tissues (upon fertilization).

The *P. berghei pm viii* gene knockout lineage developed in the mosquito midgut, but it showed a limited ability of the parasites to egress from oocysts. This was accompanied by a drastic decrease in the number of salivary gland and haemolymph sporozoites with a defect in gliding motility, leading to the block of transmission to hosts [51]. Interestingly, the egress phenotype mirrors that seen with PfPM X knockout lineages in intraerythrocytic parasites (see below). We propose that the reason piroplasms do not possess a direct homologue of the PfPM VIII/TgASP4 subclade is because they do not create—and do not need to egress from—oocysts in the tick gut tissue.

#### 2.2.3. Clade C

In recent years, Clade C of apicomplexan APs has gained a lot of attention, as this group of enzymes have been demonstrated as regulators of invasion and egress of host cells. This clade comprises *P. falciparum* plasmepsins IX and X (PfPM IX and PfPM X), and their *T. gondii* homologue TgASP3 [21,22]. Our analysis clearly identified two different isoenzymes clustering to two groups of clade C piroplasmid APs. While the first piroplasmid group (here termed *Babesia* ASP3b) firmly clusters together with PfPM X (bootstrap value of 97), and its members might thus be considered as the PfPM X direct orthologues, the sister relationship of the second piroplasmid cluster (here termed *Babesia* ASP3a) and the PM IX + TgASP3 group is not well supported (bootstrap value <50). Clade C APs are believed to undergo self-catalytic activation, upon which they proteolytically process/activate a vast number of protein precursors associated with secretory organelles of the apical complex (AC) [19,22,53]. Thus, PfPM IX and PfPM X are the major self-activating maturases standing at the top of the proteolytic machinery regulating *P. falciparum* cell invasion and egress [54]. Although the mechanism of invasion and egress employing the secretory and non-secretory parts of the AC is relatively conserved among apicomplexan parasites, species-specific alterations of the generic mechanisms have evolved [55,56]. *Babesia* and *Plasmodium* replicate solely inside host erythrocytes, and the invasion process involves initial attachment of the parasite to the host cell via a receptor-ligand-mediated interaction [57]. Both parasites interact with glycophorin receptors on the RBC surface, although their surface adhesins differ [58,59]. *Plasmodium* merozoites attach via merozoite surface proteins (MSPs), erythrocyte-binding ligands (EBLs), and reticulocyte binding-like homologues (RHs)—protein families [60] that undergo proteolytic shedding by PfPM X [53]. Proteins of the merozoite surface antigen (MSA) family that are found on the *Babesia* merozoite surface also undergo processing by a yet unidentified sheddase [61]. This role supposedly might be played by the newly identified *Babesia* ASP3b isoenzyme. Similarly, *Theileria*’s surface coat is shed by an undescribed protease, suggesting an identical role of the *Theileria* ASP3b orthologues [62]. Initial attachment of the parasite to the host erythrocyte is followed by the reorientation of the apical tip towards the host cell, and the establishment of the moving (tight) junction (MJ) [63]. The components of MJ—such as the parasite membrane-associated apical membrane antigen 1 (AMA1) and the host cell membrane-anchored rhoptry neck protein complex serving as a ligand structure—are also conserved among piroplasms [63,64]. In *P. falciparum*, AMA1 is directly cleaved by PfPM X, and subsequently shed via PfPM X-activated integral membrane serine protease subtilisin 2 (SUB2) [21]. Since AMA1 of *Babesia* parasites is also proteolytically processed at several positions [65,66,67], we propose that ASP3b might be analogously involved in AMA1 maturation. However, this concept needs to be experimentally validated because other proteases, such as the intramembrane-cleaving rhomboids that have been identified from *Babesia* [68], cannot be excluded as *Babesia* AMA-1 sheddases working in the same manner as shown in *T. gondii* tachyzoites [69]. Moving junction protein orthologues are most likely dispensable for the non-motile merozoites of *Theileria* that enter the host cell in any orientation via a passive process known as zippering [64,70]. Thus, processing of *Theileria* AMA1 via the *Theileria* ASP3b homologues might be crucial in different motile invasive stages [71]. Upon its internalization within the host cell, *P. falciparum* remains surrounded by parasitophorous vacuole membrane (PVM) and undergoes schizogony. Later, exoneme-specific serine protease subtilisin 1 (PfSUB1), which is cleaved both autocatalytically and by PfPM X, is in charge of PVM lysis, erythrocyte plasma membrane poration, and red blood cell rupture [72]. Only minutes prior to the merozoite’s egress from the erythrocyte, PfSUB1 is secreted into the parasitophorous vacuole (PV) space, where it cleaves the pseudoprotease serine repeat antigen 5 (SERA5)—the negative regulator of *P. falciparum* egress [72,73]—as well as other PV-resident proteins important for egress [74]. Additionally, PfSUB1 initiates the primary processing of merozoite surface protein complex (MSP1/6/7) [75,76]. Piroplasmid genomes encode for a single subtilisin-like protease that is believed to represent the direct orthologue of PfSUB1. This protease presumably undergoes analogous post-translation processing during secretory transport [77]. However, the analogy in identical protein substrate cleavage by *Plasmodium* and piroplasmid SUB1 proteases [78], as well as the involvement of the activated SUB1 enzyme in *Babesia* and *Theileria* parasite egress from erythrocytes, remains to be experimentally confirmed. Notably, PfPM X mRNA is also transcribed in gametocytes, while the protein can be found in gametes, zygotes, and ookinetes. In the late stage of ookinete development, PfPM X cleaves the cell-traversal protein of ookinetes and sporozoites (CelTOS) [21]. This processing is necessary for ookinetes to pass through arthropod cells to the site of oocyst formation. Moreover, the disruption of CelTOS abolishes liver infection by *P. berghei* sporozoites [79]. As CelTOS is conserved among apicomplexan parasites [80], we propose that its processing by clade C APs plays an important role during the transmission of piroplasmid species through their tick vectors.

PfPM IX, the second *P. falciparum* protease member of the apicomplexan clade C APs, has been previously confirmed to be a key maturase involved in erythrocyte invasion by *P. falciparum* merozoites [21]. However, more recent contributions have speculated as to its multifaceted role throughout the malaria parasite life cycle [53]. PfPM IX processes rhoptry-associated protein 1 (RAP1) and rhoptry neck protein 3 (RON3), which are released into the PV during invasion and later aid the parasite’s development within the PV [81,82]. The translation initiation of PM IX has been suggested during *P. berghei* gamete development in mosquitos, where PM IX cleaves merozoite thrombospondin-related anonymous protein (MTRAP), enabling the release of gametes from host erythrocytes [83]. When mosquitoes were infected with PbMTRAP knockout gametocytes, the oocyst formation was aborted. In addition, these gametocytes were unable to form ookinetes (the motile form of zygote) in vitro. Particularly, TRAP is a conserved family of proteins that are involved in the gliding motility of apicomplexan parasites, and are also found in *Babesia* [68,83]. This suggests that piroplasmid APs branching together with PM IX might be involved in the development of zygotes and kinetes within tick tissues, as indicated by the expression of BmASP3 mRNA following the detachment of tick nymphs from the host (Figure 1C).

#### 2.2.4. Clade D

Clade D is the most derived group of apicomplexan APs within our phylogenetic analysis (Figure 2); it consists of distant relatives of the human β-site amyloid precursor protein-cleaving enzyme (BACE) [27]—the major beta secretase generating amyloid-β peptides in the neurons. BACE is also responsible for the generation of the amyloid-β peptides that aggregate in the brains of Alzheimer’s patients and, thus, represents a valuable target for drug development [84]. These proteases have a long C-terminal extension with a trans-membrane domain that serves for their anchoring to membranes. The most studied member of this clade is PfPM V—a *P. falciparum* endoplasmic reticulum (ER)-resident protease that has been demonstrated to cleave proteins containing the *Plasmodium* export element RxLxE/Q/D (PEXEL) motif [26]. These effector proteins are then secreted through the PV surrounding the parasite during the intracellular infection and multiplication to host erythrocytes [26,27]. PfPM V has also been shown to be essential for gametocyte development [32]. Thus, specific inhibition of PfPM V activity appears to be a highly convenient therapy targeting both the asexual and sexual stages of malaria [32,85]. Interesting findings have been made with PM V’s corresponding *T. gondii* orthologue TgASP5—a Golgi-resident AP that processes TEXEL (*T. gondii* PEXEL-like) motif containing dense-granule proteins (GRAs) [86]. Analogously to its orthologue PfPM V, TgASP5 plays an important role during intracellular parasite survival and multiplication [87], as well as modulation of host cell responses [28]. Another *T. gondii* isoenzyme, TgASP7, which also clusters within clade D, is not expressed in the tachyzoite stage, and its functional role remains unknown [17].

Our phylogenetic analysis revealed piroplasmid clade D APs to be a single subgroup that clusters alongside *Cryptosporidium* and *Plasmodium* PM V. Thus, we propose that they play a similar role in the secretory pathway of piroplasms—the cleavage of PEXEL-like containing proteins in the ER of the cell—although we expect significant differences due to the different strategy of piroplasms in persisting inside infected cells. Shortly upon host cell invasion, *Babesia* and *Theileria* parasites are surrounded by a PVM that is derived from the host cell cytoplasmic membrane, but unlike the *Plasmodium* and *Toxoplasma* PVM, it starts its disintegration after parasite internalization [31,88,89]. It has been discussed previously that the PVM breaks down either because piroplasms are not able to incorporate lipids into the PVM during parasite intracellular growth, or because they have developed this strategy as a more convenient way to transport effector proteins into infected host cells in order to alter their morphology and physiology [90]. The persistence of piroplasms directly in host cell cytoplasm is thus different to *P. falciparum* residing in the PV surrounded by the PVM. In the malaria parasite, the PEXEL-containing protein precursors are cleaved by the ER-resident PM V immediately upon their translation. This facilitates them for export to the PV, PVM, and all the way to the host cell [26]. The PEXEL-motif-containing exportome of *P. falciparum* is estimated to include ~463 proteins [26]. On the other hand, TgASP5 recognizes only several identified TEXEL-motif-harbouring proteins, but also appears to be important for the trafficking of other (non-TEXEL)-secreted proteins during intracellular infection with *T. gondii* tachyzoites [91]. Both *Plasmodium* and *Toxoplasma* use sophisticated protein transporting complexes PTEX (plasmodium translocon of exported proteins) and the MYR translocon, respectively, which are incorporated into the PVM [92,93]. Unsurprisingly, piroplasms do not encode for components of the *Plasmodium* translocon machinery [29], with the single exception of the PTEX complex protein component HSP101, which is expressed across all piroplasms [62].

The PEXEL-like motif (PLM) has also been recognized in various piroplasmid proteins. This supports the above-given hypothesis of functional analogy between the piroplasmid clade D APs and plasmodial PM V enzymes [94]. PLM has been recognized in *B. bovis* variant erythrocyte surface antigens (VESAs) [95] involved in erythrocyte adhesion and antigenic variation of the red blood cell surface—sophisticated parasite-driven mechanisms enabling the infected erythrocytes to evade host immune responses [96]. VESA1-like proteins have also been described from the genomes of other piroplasmid species—the *Babesia* sensu stricto group [97], and the *B. microti*-like group [98]—indicating that VESA processing by clade D APs might be an immunoevasive strategy shared across *Babesia* species. Small open reading frame protein families (SmORFs) and spherical body protein-2 protein family members (SBP2) also contain PLM [94]. While SmORFs are also involved in erythrocyte adhesion [95,96], SBP2 proteins concentrate under the red blood cell cytoplasmic membrane and are believed to alternate the red blood cell surface [99]. Although PLM has not yet been detected in *Theileria* [62,97], the apparent conservation of clade D APs within the phylum suggests the preserved mode of protein processing across Piroplasmida. Further studies on clade D APs should further elucidate the exact role of these enzymes in the trafficking mechanisms of *Babesia* spp. effector proteins affecting infected host cells.

#### 2.2.5. Clade E

This group of apicomplexan APs is clearly determined by our phylogenetic analysis as the sister clade to clade B, which includes PM VII and TgASP6 (Figure 2). PM VII is thus sometimes classified together with clade B PM VI and PM VIII as the transmission-stage plasmepsins [37]. PM VII is produced in the ookinetes of *P. falciparum*, supposedly upon fertilization, as PM VII is not detected in *P. falciparum* gametocytes [23]. Its role remains unknown—it has been proven to be disposable for the parasite, as the PM VII knockout had no effect on any stage of the *P. berghei* life cycle [23]. However, the authors of this study also note that redundancy exists among transmission-stage-expressed plasmepsins—and especially clade B member PM VIII, which shares a similar expression profile with PM VII, may thus compensate for its loss of function [100]. This would probably not be the case for piroplasms, which do not possess direct homologues of PM VIII, but produce a single clade E AP orthologue. If the clade B PM VI orthologues play a role in regulating sporogony in tick salivary glands, the remaining function for clade E piroplasmid APs might thus insist in parasite invasion of tick tissues upon zygote formation. This is in accordance with the obtained expression profile of BmASP6 in stages developing within the vector midgut (Figure 1). However, some role of BmASP6 and other clade E piroplasmid APs in sporogony should not be fully excluded, as BmASP6 mRNA is also present in the salivary glands of infected tick nymphs (Figure 1).

#### 2.2.6. Clade F

This is a diverse group of apicomplexan APs first identified in our 2016 contribution [16], and now once more confirmed by our current phylogenetic analysis (Figure 2). This group is represented by TgASP1 and its coccidian homologues, as well as some APs of gregarines and free-living basal Apicomplexa. TgASP1 is an enzyme associated with the secretory pathway in non-dividing cells, which re-localizes in close proximity to the nascent inner membrane complex (IMC) of daughter cells during replication [17]. However, it’s role is non-vital for *T. gondii* [101], and Haemosporida and Piroplasmida genomes apparently do not encode any of the clade F homologues as they might have lost these disposable proteases as a part of their adaptation to the parasitic lifestyle and the evolution of specific intracellular multiplication mechanisms differing from *T. gondii* endodyogeny [102].

## 3. Conclusions

**Figure 3:**
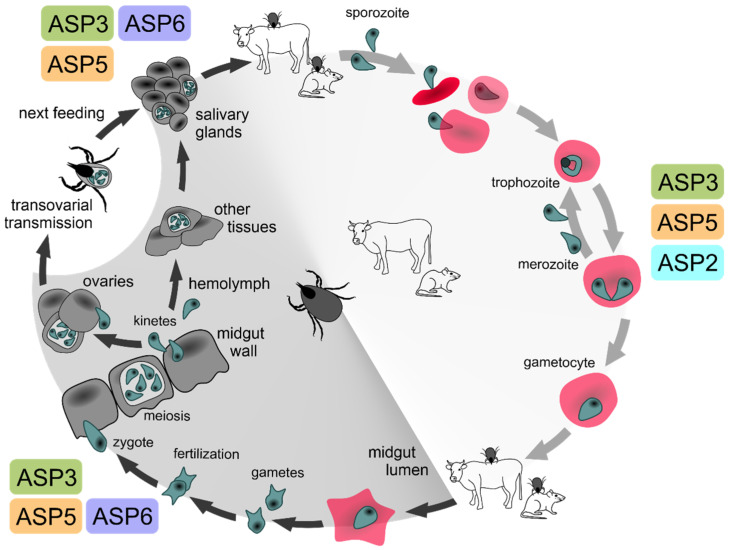
The role of *Babesia* plasmepsin-like APs marked within the generic life cycle of *Babesia* parasites. Sporozoites are transmitted from the salivary glands of an infected tick to the vertebrate host bloodstream during tick feeding. They invade red blood cells, where they start asexual multiplication (merogony). The cyclic egress and invasion of host red blood cells by haploid merozoites represents the intraerythrocytic cycle, causing the symptoms of babesiosis in vertebrates by time the first sexual stages (gametocytes) occur in the bloodstream. When another naïve tick feeds on the infected host, gametocytes are ingested with the blood meal, mature, and produce gametes in the tick gut lumen (gamogony). Newly developed gametes fuse in a zygote, which passes through the peritrophic matrix into tick gut epithelial cells. Meiotic division and subsequent mitosis give rise to primary kinetes, which invade and multiply in different tick tissues. Newly emerged secondary kinetes undergo multiplication in salivary glands (sporogony). Upon the tick metamorphosis, motile sporozoites are transmitted to the host with the blood meal (transstadial transmission). Secondary kinetes of *Babesia* sensu stricto species are capable of invading and multiplying within adult female ovaries and can be transmitted to the larval progeny (transovarial transmission), while the *Babesia microti*-like group parasites are transmitted solely transstadially [25]. The colour-marked text notes indicate the positions in the *Babesia* life cycle positions where the five aspartyl proteases ASP2, ASP3a, ASP3b, ASP5, and ASP6 supposedly play their herein-deduced roles. White background: part of *Babesia* life cycle occurring in the vertebrate host; grey background: part of *Babesia* life cycle occurring within the tick vector.

*Babesia* and other Piroplasmida encode for several pepsin (cathepsin-D-like) family AP isoenzymes. Their phylogenetic relation to malarial plasmepsins and analogous enzymes from other apicomplexan parasites enable the prediction of their various roles within the lifecycle of these erythrocyte-infecting parasites (Figure 3). These roles are associated with their different protein structures, time-expression profiles, and intracellular localization. As these enzymes have been long considered and recently validated as great therapeutic targets for malaria, they are worthy of scientific attention when proposing novel therapeutic strategies for babesiosis (piroplasmosis).

## 4. Materials and Methods

### 4.1. B. microti Propagation in Mice

*B. microti* (Franca) Reichenow (strain Peabody mjr) was obtained from ATCC (ATTC^®^ PRA-99™, USA). Two BABL/c mice, supplied by Charles River Laboratories (VELAZ), were intraperitoneally injected with 150 μL of *B. microti-*infected murine blood (50% parasitemia, 800 × 10^6^ of infected red blood cells). One mouse was kept under general anaesthesia, and the blood was collected from the carotid artery into sodium citrate-phosphate-dextrose solution (ratio 1:25, Sigma-Aldrich) on the 6th day post injection (6DPI) according to 50% parasitemia. Similarly, the blood was obtained from the second mouse when parasitemia dropped to 5% on the 10th day post injection (10DPI). The murine experiment was performed repeatedly to confirm the results of expression profiling. All laboratory animals were treated in accordance with the Animal Protection Law of the Czech Republic No. 246/1992 Sb., ethics approval No. 25/2018.

### 4.2. RNA Isolation from Tick Tissues and Murine Blood Cells

Pathogen-free *Ixodes ricinus* nymphs were obtained from the in-house breeding facility of the Institute of Parasitology, BC CAS, Ceske Budejovice, Czech Republic. Twenty individuals were placed on one *B. microti*-positive BABL/c mouse in the acute phase of infection (1–4DPI, Figure 1A) and allowed to feed until repletion (around 72 h). The fully fed (FF) nymphs were collected and surface-sterilized by washing in 3% H_2_O_2_, 70% ethanol, and distilled water (each wash 30 s). Ticks were separated into two groups: first group of 10 nymphs was dissected immediately (FF stage), while the other 10 individuals were kept at room temperature in a humid chamber until they were dissected on the 6th day post detachment (6DPD). Dissection of tick tissues (salivary glands and midguts) was performed under a stereomicroscope (Olympus) on wax dishes with diethyl pyrocarbonate (DEPC)-treated cold phosphate-buffered saline (PBS), and then transferred into RA1 buffer (NucleoSpin RNA II Kit, Macherey-Nagel, Düren, Germany) supplemented with β-mercaptoethanol (Sigma-Aldrich). Prior to the extraction, the collected tissues were homogenised using an insulin syringe. Total RNA was extracted from the pool of midguts and salivary glands originating from 10 individual ticks via the NucleoSpin RNA II Kit, following the protocol provided by the manufacturer (Macherey-Nagel, Düren, Germany). Murine blood total RNA was isolated using the previously described protocol [103]. Samples were collected from two timepoints at 6 and 10 DPI. The quality and concentration of total RNA were checked by gel electrophoresis and determined using a NanoDrop UV spectrophotometer (Thermo Fisher Scientific; Waltham, MA, USA), and RNA samples were stored at −80 °C.

### 4.3. Quantitative RT-PCR

Reverse transcription was performed from 1 µg of total RNA isolate using the Transcriptor High-Fidelity cDNA Synthesis Kit (Roche Diagnostics GmbH; Mannheim, Germany). The resulting cDNA was used as a template for the quantitative real-time PCR (qRT-PCR) using a LightCycler 480 (Roche Diagnostics GmbH), the Fast Star Universal SYBR Green Master Mix (Roche Diagnostics GmbH), and according primer pairs BmASP2 forward: 5′-TCCGGCGTCTATTGAAGAGT-3′/BmASP2 reverse: 5′-TGAACCGGTGTCAAAAACAA-3′; BmASP3 forward: 5′-GGAAGCTTGGGGAGTCTGTA-3′/BmASP3 reverse: 5′-TGTGCTCCCTGTGTCGAATA-3′; BmASP5 forward: 5′-GCCCAAACACCACCAACTAT-3′/BmASP5 reverse: 5′-CACCAAATGCGAGATACACG-3′; BmASP6 forward: 5′-GATTGGGCTTCCCAAACAC-3′/BmASP6 reverse: 5′-ATCCGCCAGTTGAATCTTTG-3′. All qRT-PCR amplifications were performed in technical triplicates. Relative expressions were calculated using the mathematical model of the ΔCt method [104] and normalized to *B. microti* actin (GenBank XM_012791652; primers—BmActin forward: 5′-GGCCTACTCACAGCCCTTTA-3′/BmActin reverse: 5′-ACAGGGTTGTAGAGTGTTGGTT-3′). To express the representation of all isoenzymes as a percentage per time point, the ASP with the highest Ct value was set as 100%, and the values for other ASP isoenzymes were accordingly recalculated. The algorithm applied later adjusted the values to fit the total of 100% in a pie chart.

### 4.4. Phylogenetic Analysis

The dataset used for the phylogenetic analysis comprised 106 AP protein sequences of representatives from the phylum Apicomplexa, and related *Vitrella* and *Chromera* spp. Clade F involving digestive plasmepsins served as an outgroup. All sequences were retrieved from either GenBank or EuPathDB using the blastp and tblastn BLAST algorithms and an E-value cutoff of 10^−5^. Alignment was constructed in Geneious Prime 2020.1.2. using MAFFT v7.017 [105] with the default parameters for the gap opening penalty (1.53) and the offset value (0.123). The protein sequences were crosschecked for the presence of DTG/DTG or DTG/DSG aspartic protease motifs. Poorly aligned N- (signal peptide included) and C-termini were manually trimmed, which resulted in the final alignment comprising 324 amino acid positions. The phylogenetic tree was reconstructed via maximum likelihood (ML) method in IQ-TREE v1.6.12 [106], using the WAG + F + I + G4 model selected by ModelFinder [107]. Bootstraps were based on 1000 replicates. The tree was visualized in Geneious Prime v2019.0.4 and graphically modified in CorelDRAW graphic suite 2020.

## Data Availability

All data are either contained within the manuscript and supporting information or available from the corresponding author on reasonable request.

## Supplementary Materials

The multiple alignment data used to construct the maximum likelihood tree in Figure 2 are accessible online at Mendeley Data, link: http://dx.doi.org/10.17632/ds3f2j32ny.1, accessed on 23 September 2021.
